# Antibacterial Prodrugs to Overcome Bacterial Resistance

**DOI:** 10.3390/molecules25071543

**Published:** 2020-03-28

**Authors:** Buthaina Jubeh, Zeinab Breijyeh, Rafik Karaman

**Affiliations:** Pharmaceutical Sciences Department, Faculty of Pharmacy, Al-Quds University, Jerusalem P.O. Box 20002, Palestine; bjubeh@gmail.com (B.J.); z88breijyeh@gmail.com (Z.B.)

**Keywords:** prodrugs, biotransformation, targeting, β-lactam antibiotics, β-lactamases, pathogens, resistance

## Abstract

Bacterial resistance to present antibiotics is emerging at a high pace that makes the development of new treatments a must. At the same time, the development of novel antibiotics for resistant bacteria is a slow-paced process. Amid the massive need for new drug treatments to combat resistance, time and effort preserving approaches, like the prodrug approach, are most needed. Prodrugs are pharmacologically inactive entities of active drugs that undergo biotransformation before eliciting their pharmacological effects. A prodrug strategy can be used to revive drugs discarded due to a lack of appropriate pharmacokinetic and drug-like properties, or high host toxicity. A special advantage of the use of the prodrug approach in the era of bacterial resistance is targeting resistant bacteria by developing prodrugs that require bacterium-specific enzymes to release the active drug. In this article, we review the up-to-date implementation of prodrugs to develop medications that are active against drug-resistant bacteria.

## 1. Introduction

Nowadays, the issue of pathogens resistant to drugs and the urgent need for new compounds that are capable of eradicating these pathogens are well known and understood. Most of the resistance mechanisms by bacteria have been discovered and described, such as enzymatic degradation, target modification, overexpression of efflux pumps and decreased uptake. Multidrug-resistant bacteria show resistance to three or more antibiotic classes, and are a major cause of mortality as indicated by the World Health Organization (WHO) [1]. 

Penicillin was discovered in the 1940s, and shortly after, resistance was developed. By the 1950s, many β-lactam antibiotics were discovered; however, resistance was identified in the 1960s. Later on, fluoroquinolones were introduced to treat Gram-negative bacteria in the 1980s, but resistance was quickly developed due to chromosomal mutations. Antibiotic discovery continued over decades, and millions of drugs were introduced to the market; this has led to less expensive antibiotics and the irresponsible dispensing of antibiotics without prescriptions, with the absence of regulatory guidelines in many countries. This has all contributed to the development of resistant strains (Table 1) [2,3].

The introduction of a new drug to the market generally takes 10–15 years, with a huge budget, and most of the developed drug candidates fail the clinical stages due to their poor pharmacokinetic properties. Therefore, alternative and novel approaches to develop new drugs, especially antibiotics, should be established urgently. Most of the strategies that are being used today are based on prodrugs and the design of new molecules, but the latter process has disadvantages in that it is expensive and time-consuming. The prodrug approach is more efficient and cheaper, and about a tenth of marketed drugs are classified as prodrugs [4].

In this review, we focus on newly developed antibiotic prodrugs and their discovery to fight the dilemma of the century: antibiotic resistance.

## 2. Prodrugs

**Figure 1:**
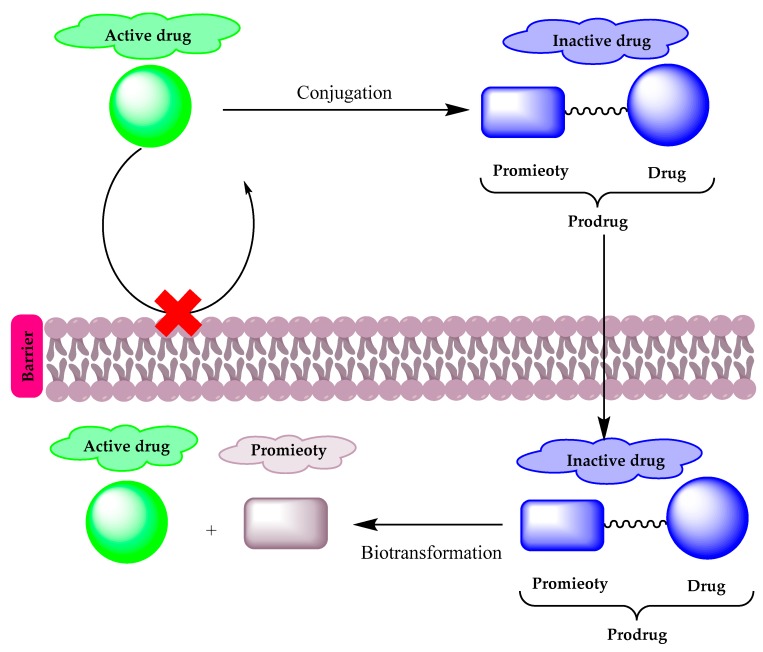
A diagram illustrating the prodrug concept.

**Figure 2:**
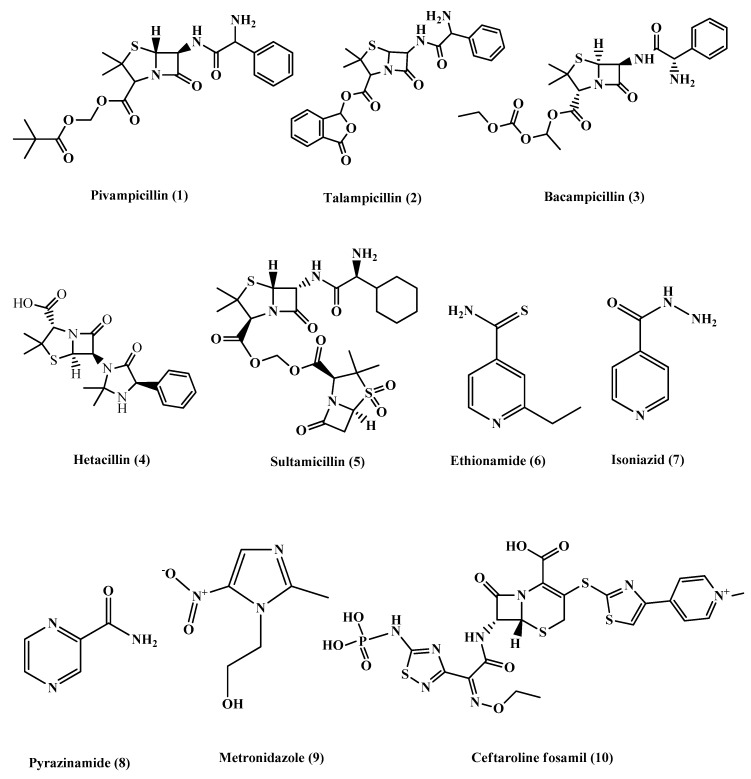
Chemical structures of pivampicillin, talampicillin, bacampicillin, hetacillin, Sultamicillin, ethionamide, isoniazid, pyrazinamide, metronidazole and ceftaroline fosamil.

Prodrugs are pharmacologically inactive compounds that inter- or intra-convert inside the body into active forms and non-toxic moieties by enzymatic or chemical reactions. In prodrug design, the addition or removal of certain parts of the parent drug can alter its bioavailability, absorption, and permeability without affecting its pharmacological activity. Prodrugs are classified into three types:(1) carrier-linked prodrugs in which the active drug is linked to a promoiety, which is removed by an enzymatic or chemical reaction to release the active drug (Figure 1),(2) bioprecursor prodrugs in which a molecular modification is made to the active drug and that can undergo molecular modification by oxidation or reduction reactions to release the active drug, and finally (3) double prodrugs that are attached to each other by two linkers that can be cleaved by different mechanisms, such as a co-drug, in which two biologically active drugs are linked in a single molecule.

The prodrug approach is now very popular, and it is not just used to alter the physicochemical parameters but is also used to alter many molecular and cellular factors such as influx/efflux membrane transporters and the expression/distribution of cellular proteins, in addition to drug targeting and delivery to enhance treatment and therapeutic efficacy [4,5,6,7,8,9].

Most of the β-lactam antibiotics are poorly absorbed, and the prodrug approach was used to improve their oral bioavailability.Pivampicillin **(1)** (Figure 2) is an ampicillin derivative that is considered one of the first prodrugs to be developed, which is enzymatically hydrolyzed by esterases to give the active drug ampicillin, with inactive pivalic acid. Moreover, talampicillin **(2)**, bacampicillin **(3)** andhetacillin **(4)** (Figure 2) are all ester prodrugs that were developed to improve ampicillin bioavailability. Another form of prodrug is made by attaching a linker to the active drug to combine it with other antibiotics or different drugs such as sultamicillin **(5)** (Figure 2), which is a prodrug that links ampicillin and sulbactam by a methylene group [10,11,12,13]. Other prodrugs were developed to improve the activity of existing antibiotics including ethionamide **(6)**, isoniazid **(7)** and pyrazinamide **(8)** (Figure 2), used to treat *Mycobacterium tuberculosis*. In addition, metronidazole **(9)** (Figure 2) acts as a prodrug, which is used for the treatment of *Helicobacter pylori* and anaerobic infections. Metronidazole requires reduction by specific enzymes to be activated; the redox intermediate could be responsible for killing the microorganisms by targeting intracellular components such asthe bacterial cell membrane, RNA, DNA and proteins. Resistance to Metronidazole has been reported especially in *H. Pylori*, but its mechanism of action is not well known [1,14].

**Figure 3:**
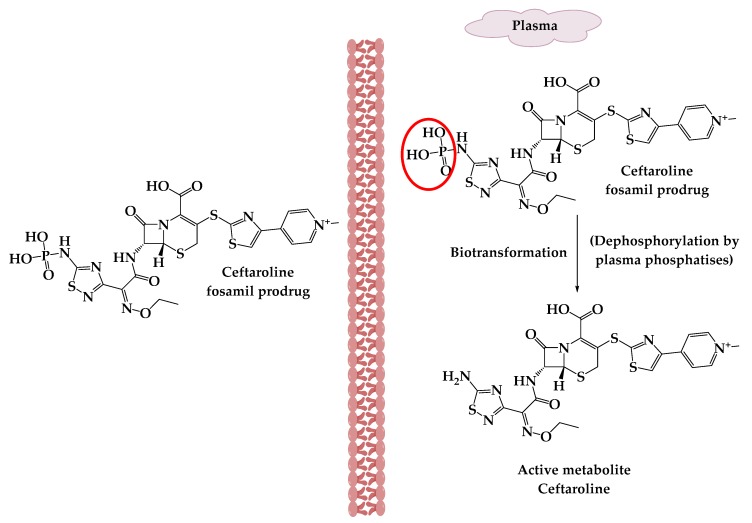
The activation of ceftaroline fosamil prodrug.

Ceftaroline fosamil **(10)** (Figure 2) is an example of cephalosporin prodrugs that upon activation give the active metabolite ceftaroline, which acts by binding to penicillin-binding proteins that inhibit bacterial cell wall synthesis (Figure 3).Ceftaroline fosamil has potent activity against the bacteria causing community-acquired bacterial pneumonia (CABP), such as multidrug-resistant *Streptococcus pneumoniae*(MDRSP), penicillin-resistant *Streptococcus pneumonia* (PRSP) and methicillin-resistant *Staphylococcus aureus* (MRSA) [15].

As we mentioned earlier, most of the antibiotics that have been developed over the decades encountered bacterial resistance, which formed a wall in front of research and caused large pharmaceutical companies to abandon already-registered antibiotics [2].

## 3. Prodrug Applications against Resistant Bacterial Pathogens

In combating antibiotic resistance, prodrugs have been applied either to revive antibiotics that possess activity against resistant pathogens—but that cannot be used clinically because of their suboptimal pharmacokinetics—or as a targeting drug tool to overcome the resistance barriers and minimize host toxicity. Herein, we describe the recently reported antibiotic prodrugs for which the prodrug approach was the key to fight resistance.

### 3.1. A β-Lactamase-Activated Ciprofloxacin Prodrug

The most important and prevalent determinant of antibiotic resistance is the expression of β-lactamase enzymes, which hydrolyze the β-lactam antibiotics, preventing their interaction with the penicillin-binding proteins, their therapeutic targets. Extended-spectrum β-lactamases that have the ability to cleave a wide range of β-lactam antibiotics, such as the CTX-M class (extended-spectrum β-lactamases active on CefoTaXime, first isolated in Munich), are of particular concern [16,17,18]. Broad-spectrum antibiotics, like ciprofloxacin, are increasingly used to treat infections caused by β-lactam-resistant bacterial infections, especially *Escherichia coli*-caused urinary tract infections [19]. The problem is that the use of broad-spectrum antibiotics disrupts the microbiota, the beneficial gastrointestinal bacteria, which normally lead to secondary infections caused by other antibiotic-resistant bacteria [20,21]. 

A novel cephalosporin-fluoroquinolone prodrug was developed by Evans et al. [22]. The prodrug was designed to selectively deliver a broad-spectrum antibiotic, ciprofloxacin, to only β-lactamase expressing bacteria, while having minimal effects on bacteria that do not express this feature of resistance, the β-lactamase enzymes. In this approach, it was meant for the prodrug motif to enable substrate turnover rather than inhibit the β-lactamase enzyme. A cephalosporin core was selected as the β-lactam component, as cephalosporins are well-known β-lactams efficiently hydrolyzed by β-lactamases. Generally, cephalosporins are considered good prodrug moieties because they allow the bioreversible modification of other active drugs by the conjugation of one of their functional groups at the 3′-position of the cephem core. 

**Figure 4:**
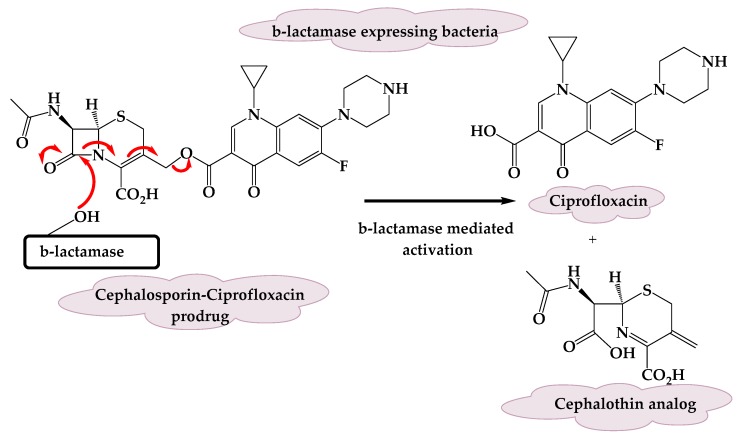
Mechanism of activation of the cephalosporin-ciprofloxacin prodrug.

To achieve the desired selectivity profile, ciprofloxacin was attached via the carboxylic acid to give the 3′-cephem ester. To reduce the antibacterial activity of the intact prodrug, a program of optimization was undertaken by a modification to the cephalosporin portion. A cephalosporin analog with a methyl group at the α-position relative to the amide carbonyl of the reference compound cephalothin gave the best results. Overall, the prodrug exhibited a β-lactamase-mediated intracellular release of ciprofloxacin upon cleavage of the cephalosporin (Figure 4), selectivity toward β-lactamase-expressing *E. coli*, no activity on non-β-lactamase expressing bacteria, and very little activity of the intact prodrug [22]. This strategy of prodrug application opens the gate for using broad-spectrum antibiotics to treat resistant pathogens in a selective manner.

### 3.2. Cephalosporin-3-Diazeniumdiolate

**Figure 5:**
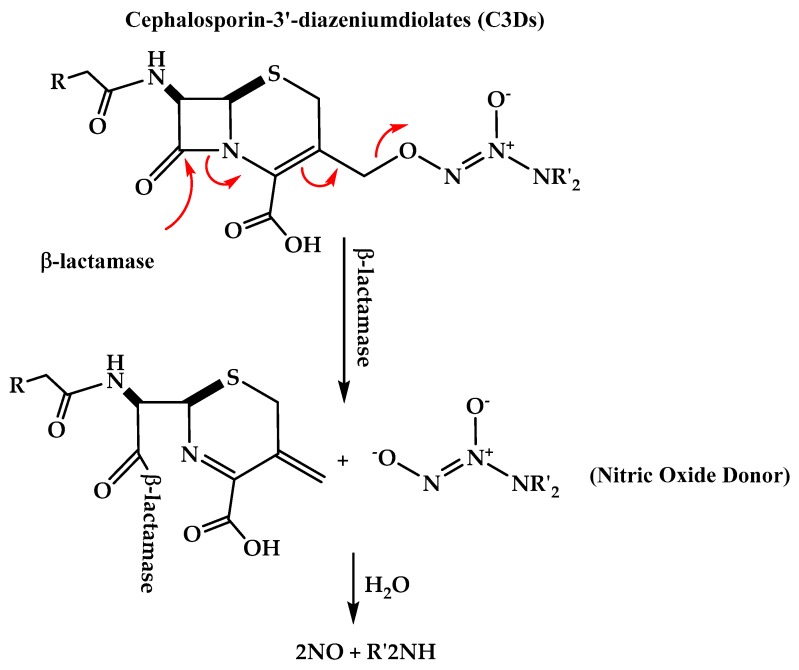
Mechanism of action of the nitric oxide donor prodrug cephalosporin-3′-diazeniumdiolates (C3Ds).

**Figure 6:**
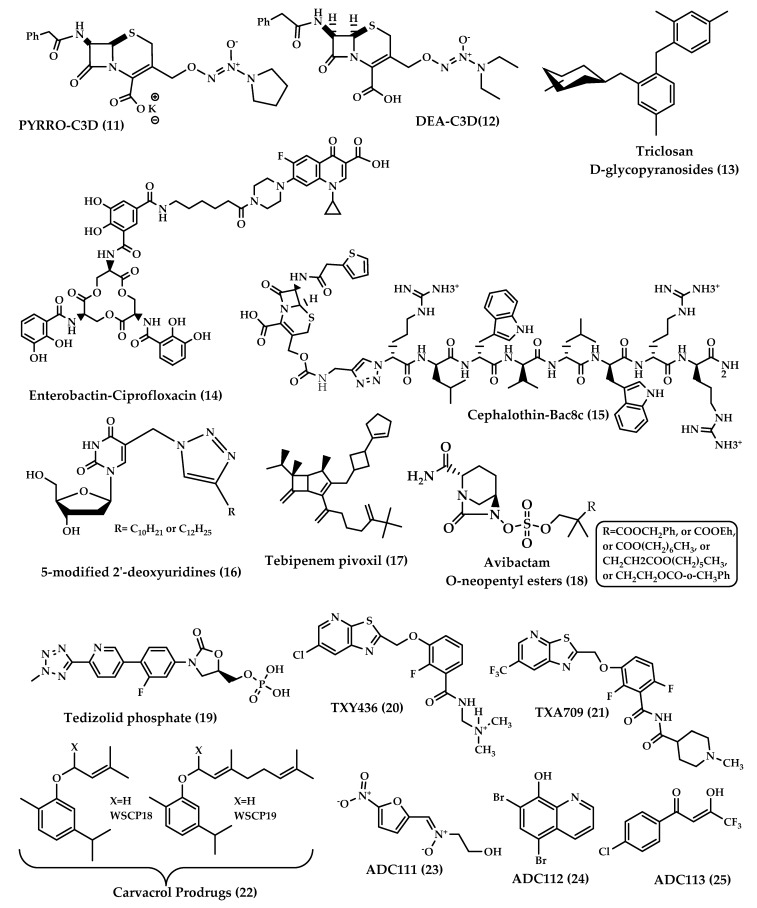
Chemical structures of **11**–**25**.

Cephalosporin-3′-diazeniumdiolates (C3Ds) are a new class of nitric oxide (NO) donor prodrugs. These prodrugs have a β-lactam ring in their structures and are designed to selectively deliver NO to bacterial infection sites after the reaction with β-lactamases and the cleavage of β-lactam by transpeptidase (Figure 5) [23,24]. Currently, there are some C3Ds that are under development, including PYRRO-C3D (pyrro-cephalosporin-3′-diazeniumdiolate) **(11)**, and DEA-C3D(diethylamin-cephalosporin-3′-diazeniumdiolate) **(12)** (Figure 6). DEA-C3D, the prototypical example of a C3D, contains the phenacetyl side chain of cefaloram, a first-generation cephalosporin, and the diazeniumdiolate NO donor. In vitro studies have shown that DEA-C3D was able to disperse biofilms formed by multiple clinical isolates of *Pseudomonas aeruginosa*, and that when combined with colistin, it caused the near-complete eradication of *P. aeruginosa* biofilms [25]. 

Another representative of C3Ds is PYRRO-C3D, which contains the diazeniumdiolate NO donor PYRRO-NO. PYRRO-C3D undergoes β-lactam cleavage by transpeptidases. Transpeptidase-reactive C3Ds have a dual action in which they act as NO-mediated anti-biofilm agents and possess intrinsic β-lactam-mediated antibacterial effects [23]. It was found that PYRRO-C3D can reduce the viability of planktonic and biofilm pneumococci in the absence of β-lactamases. A study that was done to test the activity of PYRRO-C3D against a non-typeable *Haemophilus influenza* (NTHi) biofilms showed that PYRRO-C3D enhanced the efficacy of azithromycin against NTHi biofilms and can act as a promising adjunctive treatment for reducing biofilm-associated antibiotic tolerance [23,26].

### 3.3. Triclosan Glycoside Prodrugs

The idea of using glycoside derivatives of antibacterials as bacterium-targeting prodrugs came about as a result of the discovery of glycosidase enzyme expression in bacteria [27,28]. Triclosan is an antibacterial and antifungal agent that has been used as a disinfectant. Triclosan acts by inhibiting fatty acid synthesis, and in high concentrations, it disrupts the cell wall [28]. This agent is used only topically due to its low solubility at physiological pH. It was expected that glycoside derivatives on the hydroxyl group of triclosan would enhance bacterial uptake by active transport. Glycoside derivatives of triclosan (α-D-glycopyranosides and β-D-glycopyranosides) **(13)** (Figure 6) have the ability to inhibit the growth of Gram-positive and Gram-negative bacteria and showed potent, selective antibacterial activity and increased aqueous solubility compared to triclosan. This earned them the advantage to be used orally for the treatment of systemic infections [29].

### 3.4. Enterobactin-Antibiotic Conjugates

**Figure 7:**
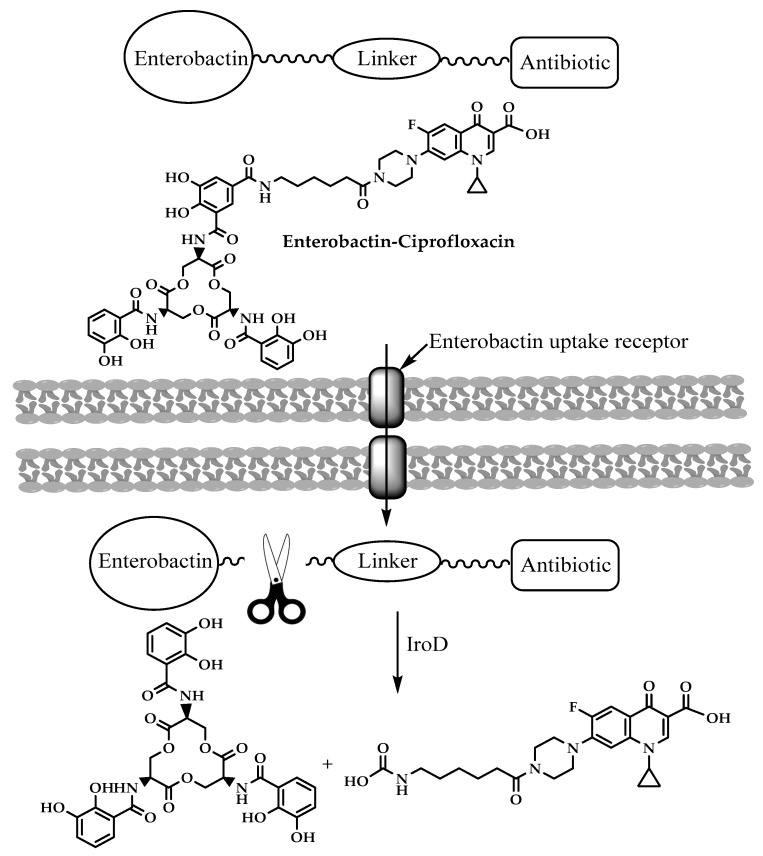
Enterobactin-ciprofloxacin conjugates activation.

The prodrug approach has been used to attain drug targeting with certain antibiotics. Zheng and Nolan [30] presented a strategy to achieve intracellular antibiotic targeting, as well as pathogen-specific activity, by making siderophore-antibiotic conjugates. This strategy is based on linking antibiotics with enterobactin—a siderophore that has receptors on bacterial surfaces and is responsible for the uptake of iron—generating inactive prodrugs. This linkage enables the uptake of the siderophore along with the antibiotic linked to it, and then the hydrolysis of the conjugate and the activation of the antibiotic take place in the bacterial cytoplasm, involving specific cytoplasmic enzymes [31]. Enterobactin (Ent) is a tricatecholatesiderophore (iron carrier) naturally produced by enteric Gram-negative bacteria like *E. coli*, and is secreted in the host vertebrate to acquire iron. Recently, enterobactin-ciprofloxacin conjugate **(14)** (Figure 6) discovery was reported by Neumann et al. [30]. Ent–Ciprofloxacin is a conjugate that has ciprofloxacin attached to an alkyl linker at one of the catechols of Ent; the result is an inactive prodrug of ciprofloxacin that is guided into the cytoplasm by Ent uptake machinery (Figure 7). Intracellularly, the prodrug is activated by the cytoplasmic esteraseIroD, an enzyme that is only expressed by *E. coli* that express the iroA gene cluster—a pathogen-associated cluster—which makes the prodrug selective for *E. coli* that have the iroA gene cluster, while having no activity against nonpathogenic clusters [30].

Ent–Ciprofloxacin was not the first Ent to be used for intracellular delivery in *E. coli*. Ent conjugation was tried earlier on the β-lactams ampicillin and amoxicillin. Ent–amoxicillin/ampicillin conjugates exert antibacterial activity after being recognized by outer membrane-siderophore receptors and transported into *E. coli* via the outer membrane transporters FepA and IroN. The prodrugs had antimicrobial activity enhanced up to 1000-fold, relative to the parent drugs, against uropathogenic *E. coli*, and selectively kill uropathogenic *E. coli* that express the IroN receptor [31,32]. 

### 3.5. Antimicrobial Peptide Prodrugs

Antimicrobial peptides (AMPs), also known as host defense peptides, are peptidic molecular mediators of innate immunity found in multicellular organisms that have antimicrobial activity [33,34]. AMPs exert direct microbicidal activity by having an amphipathic and cationic nature that enables them to be inserted in microbial cytoplasmic membranes, increasing permeation and causing cell lysis [34,35,36]. Interests in the development of AMP antibiotics, especially against resistant bacteria, are increasing [37,38,39,40,41], but unwanted cell toxicity forms a major limitation for their improvement. The prodrug strategy is a promising solution to solve the toxicity problem and fulfill bacterial selectivity, by making AMP prodrugs that permit the cationic feature to be transiently reduced by the reversible conjugation of an anionic promoiety and that can be activated by specific bacterial enzymes [42]. 

This strategy was applied in a study in which two prodrugs of the AMPs, P18 and WMR (W and R modified myxinidin peptide), were developed. P18 is a hybrid of cecropin and magainin, two innate immunity peptides from the moth Hyalophoracecropia and the frog Xenopuslaevis, respectively. WMR is a myxinidin analog from the hagfish Myxineglutinosa. The prodrugs were prepared by amidation at the parent P18 and WMR C-termini and the elongation of the N-termini with the residual amino acid AAG motif, from the neutrophil elastase sensitive linker, to give the pro-peptides (prodrugs) pro-P18 and pro-WMR, of the sequencesAc-EEEEAAAGkwklfkklpkflhlakkf-NH2 and Ac-EEEEAAAGwglrrllkygkrs-NH2 respectively (uppercase letters denote L-amino acids and lowercase letters denote D-amino acids, Ac- denotes N-terminal acetylation, and -NH2 denotes C-terminal amidation). The study described the action of the two AMPs and their prodrugs on models of bacterial and mammalian cells using the membrane permeabilization assay. It was found that the pro-peptides exerted a broad-spectrum antibacterial effect which was associated with membrane disruption. Both pro-peptides induced similar and potent permeabilizations of the bacterial model liposomes. As for the effect on the human model liposome, pro-P18 was 9-fold more lytic than pro-WMR. Substantially, the on-target selectivity between bacterial and human membranes was improved, reducing the toxicity against human membranes for both candidate peptides. This prodrug strategy suggests that the target selectivity of AMP can be improved by the near-complete neutralization of the AMP’s net charge, which is likely to reduce their propensity to affect biological membranes [42].

Another implementation of the prodrug approach to impart selective targeting for AMPs was the synthesis of β-lactam-AMP conjugates: prodrugs in which AMPs are conjugated with cephalosporins to trigger selectivity by bacterial β-lactamases. The binding between the β-lactam core of the prodrug and the β-lactamase enzyme will trigger the release of the active AMP inside the resistant, β-lactamase expressing, bacteria. Cephalothin-Bac8c **(15)** (Figure 6) is a conjugate that links cephalothin, a first-generation cephalosporin, with Bac8c—an AMP derived from the bovinedodecapeptidebactenecin [43]—through a carbamate-1,4-triazole linker. Cephalothin-Bac8c is a candidate for β-lactam-AMP prodrug conjugates with promising antibacterial activity targeting resistant extended-spectrum β-lactamase producing *Enterobacteriaceae* [44].

### 3.6. Prodrugs of 5-Modified 2ʹ-Deoxyuridines

Pyrimidine nucleoside derivatives with a lengthy substituent at the C-5 position of the nucleobase were shown to have in vitro antimicrobial activity [45,46]. The mechanism of action is unclear, but it was shown that some derivatives selectively inhibit the microorganismic enzyme flavin-dependent thymidylate synthase (ThyX), an enzyme that is absent in mammals. Other derivatives demonstrated destruction of the mycobacterial cell wall [47].

Negryaet et al. [48] have recently synthesized a set of pyrimidine nucleoside derivatives. The nucleoside derivatives showed antitubercular activity. Two compounds of the derivatives had significant activity against *M. tuberculosis* resistant strains; the compounds are 5-dodecyloxymethyl 2ʹdeoxyuridine and 5-[4-decyl-(1,2,3-triazol-1-yl) methyl]-2ʹdeoxyuridine. Although the nucleoside derivatives showed high activity, their water solubility was low because of the presence of bulky lipophilic substituents. The poor water solubility made it troublesome to study the antibacterial activity of the nucleoside derivatives on different bacteria. The prodrug approach was used to enhance the water solubility of the 5-C substituted nucleosides. Carrier-linked prodrugs of 5-modified 2ʹ-deoxyuridines **(16)** (Figure 6) were synthesized by Negryaet et al. [48] using a carbonate group to link a triethylene and tetraethylene glycol moiety to the 3′- and 5′-hydroxyl groups of the parent compounds. The obtained compounds were at least two orders of magnitude more soluble than their parent drugs, with a hydrolysis time of 5–12 h. The prodrugs showed moderate activity against some Gram-positive bacteria and low cytotoxicity towards mammalian cells. Their activity included the killing of resistant strains of *S. aureus* and *Mycobacteriumsmegmatis* [48].

### 3.7. The Tebipenempivoxil Prodrug

Tebipenem (SPR859) is a β-lactam antibiotic belonging to the carbapenem family and is active against Gram-negative and Gram-positive pathogens, but its high hydrophilicity limits its oral absorption. Tebipenempivoxil **(17)** (Figure 6) is an orally-administrated pivaloyloxymethyl ester prodrug of tebipenemwith better absorption and high bioavailability, which is currently approved only in Japan as a granule formulation for the treatment of ear, nose, throat and respiratory infections in pediatrics. Tebipenempivoxil HBr salt is currently under development for the treatment of complicated urinary tract infections in adults. Pharmacokinetics and pharmacodynamics activity showed that tebipenempivoxil HBr is a good alternative oral treatment for resistant Gram-negative pathogens and may serve as a new antibacterial agent [49].

### 3.8. Avibactam Prodrugs

β-lactamase inhibitors restored the effectiveness of β-lactam antibiotics. None of the β-lactamase inhibitors other than clavulanic acid are orally available. This is the case of avibactam, which is a potent diazabicyclooctane inhibitor of a wide spectrum of β-lactamases but lacks the proper oral bioavailability. Gordon et al. [50] have reported the synthesis and testing of avibactam O-neopentyl ester prodrugs **(18)** (Figure 6) designed to mask the charged sulfate moiety in avibactam’s structure, a moiety that causes a major obstacle for oral absorption. Coupled with anappropriate antibiotic, avibactam has the potential to treat serious Gram-negative infections without the need for intravenous injections [50].

### 3.9. Tedizolid Phosphate (TR701)

Tedizolid phosphate **(19)** (Figure 6) is an orally absorbed phosphate prodrug of tedizolid (TR700): an antibiotic of the new class oxazolidinones. Among this class, linezolid is the only marketed oxazolidinone. Oxazolidinone antibiotics are protein synthesis inhibitors that bind to the 50S ribosome and prevent the formation of the 70S complex [51]. Oxazolidinones are unlikely to have cross-sensitivity with other antibiotics because of a unique site of action that is the ribosomal peptidyltransferase center [52].

Tedizolid was found to be 4–8 fold more potent than linezolid. It is provento be active against methicillin-sensitiveand methicillin-resistant *Staphylococcus aureus*, various strains of *Enterococcusfaecalis* and *Streptococcus spp*. More importantly, it had activity against linezolid-resistant isolates; this activity and potency are suggested to happen due to additional binding site interactions [53,54]. All of this demonstrates that tedizolid phosphate could be a new treatment for multidrug-resistant Gram-positive bacteria.

### 3.10. FtsZ-Targeting Benzamide Prodrugs

Fts-Z (Filamenting temperature-sensitive mutant Z) is a prokaryote-specific protein involved in bacterial cell division; this protein represents a new antibiotic target. The compound PC190723 was among the first FtsZ-Targeting Benzamides to be proved effective against methicillin-sensitive and resistant *Staphylococcus aureus* (MSSA and MRSA). The poor pharmacokinetics and drug-like properties of PC190723 hindered its clinical development, but the development of the N-Mannich base prodrug TXY436 **(20)** (Figure 6) followed, which exhibited oral bioavailability and efficacy, as well as enhanced intravenous efficacy [55].

**Figure 8:**
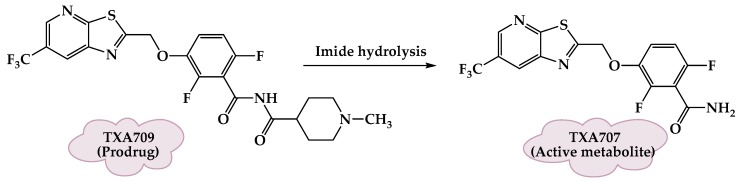
The activation of TXA709 prodrug to the active metabolite TXA707.

Despite the enhanced pharmaceutical properties of TXY436, it still required high efficacious doses. A new prodrug TXA709 **(21)** (Figure 6) was designed, based on TXY436, to enhance the metabolic stability by having a CF_3_ functionality replacing the Cl groups on the pyridyl ring. The prodrug TXA709 is rapidly converted in vivo to TXA707: an active, benzamide-derivative, FtsZ-Targeting compound (Figure 8). As a result, TXA709 has a 6.5-fold-longer half-life and a 3-fold-greater oral bioavailability relative to TXY436, a potent bactericidal activity against resistant *S. aureus* strains, and minimal toxicity towards mammalian cells [56].

### 3.11. Carvacrol Prodrugs

Carvacrol is a natural monoterpene; it is a component of phenolic essential oils particularly abundant in plants belonging to the Lamiaceae family. Carvacrol is a compound with emerging potential for application in microbial infection management because of its antimicrobial activity. Biofilm formation is one mechanism by which bacteria have developed resistance to drugs. Carvacrol can inhibit the growth of bacterial biofilms and interfere with biofilm formation, hence carvacrol has recently attracted much attention [57].

Carvacrol has an amphipathic structure that allows it to spread through the polar matrix of bacterial biofilms and to disrupt bacterial membranes; it increases the fluidity, permeability, and perturbation of the cytoplasmatic membranes. Carvacrol acts on biofilms produced by Gram-positive bacteria, especially those produced by *S. aureus* and *Staphylococcus epidermidis*. It disintegrates the outer membrane of Gram-negative bacteria as well [58].

The poor physicochemical properties of carvacrol like low water solubility, low chemical stability and high volatility restrain its potential therapeutic uses. These disadvantages can be overcome by the prodrug approach. Recently, 23 carvacrol prodrugs were synthesized and evaluated for their activity against selected Gram-positive and Gram-negative bacteria. Hydrophilic carvacrol prodrugs, prepared using polar natural groups like amino acids, showed increased water solubility. Lipophilic prodrugs were obtained by the prenylation of the hydroxyl group of carvacrol. Two lipophilic prodrugs (WSCP18-19) **(22)** (Figure 6) showed the most promising results, with antibacterial and anti-biofilm activities against *S. aureus* and *Staphylococcus epidermidis*. Moreover, the prodrugs exerted increased stabilities in human plasma and simulated fluids. Additionally, all of the obtained prodrugs were nontoxic to mammalian cells [58].

### 3.12. ADC111, ADC112 and ADC113

The need for broad-spectrum antibiotics can be met by the development of prodrugs that are activated by enzymes that are bacterium-specific, to give reactive compounds that could kill persisters and accumulate over time. Hence, a screen of 55,000 compounds of prodrugs was done by Fleck et al. [59]. The screen was directed to prodrugs that have been discarded in conventional high-throughput screening campaigns due to the lack of specificity of the mechanism of action. The screen was based on identifying compounds that nonspecifically inhibit the reduction of alamarBlue, a viability dye, and then testing for cytotoxicity and eliminating generally-toxic compounds. Twenty hit compounds were active against *E. coli*. Out of the 20 hits, three prodrugs were further developed: ADC111 **(23),** ADC112 **(24)** and ADC113 **(25)** (Figure 6).

ADC111 is an analog of the nitrofuran prodrug, nitrofurantoin, an antibacterial that is used to treat urinary tract infections [60]. ADC111′s activity depends on the presence of the activating nitroreductase enzymes. ADC111 was considerably more active against *E. coli* and less toxic against mammalian cells compared to its analog nitrofurantoin. Its activity was greater against growing cells than against persister cells [59]. ADC112 is an analog of the antimicrobial tilbroquinol, and its mechanism of action is unknown [61]. ADC112 achieved the complete sterilization and killing of even the stationary cells of *E. coli*. This prodrug was the most potent one against *E. coli* [59]. ADC113 is a compound with a di-ketone functionality that does not belong to an approved antimicrobial class. ADC113 had excellent killing activity against growing *E. coli* cells and exerted good killing of stationary cells. Its killing in biofilms was comparable to ciprofloxacin. The three prodrugs had a good spectrum of antimicrobial activity and showed good-to-excellent activity against biofilms and stationary cultures [59].

## 4. Conclusions

The emergence of bacterial pathogens that are resistant to antibiotics and the shortage of conventional antibiotics to treat infections associated with resistant bacteria have created an urgent need for new treatment options. Unfortunately, bacteria produce and acquire genetic changes to support their survival. Enzymatic degradation, target modification, the overexpression of efflux pumps and decreased drug uptake are all mechanisms exerted by bacteria to obtain treatment resistance. Moreover, chronic infections are characterized by the accumulation of bacterial persisters in biofilms, shielding them from the immune system and conventional antibiotics. The success of the prodrug approach in resolving the limitations of drugs in a relatively short time has encouraged research into antibiotic prodrugs, a strategy that will enable the eradication of resistant bacteria by upgrading the existing antibiotics. One of the major determinants of resistant bacteria is their expression of β-lactamase enzymes; this feature was exploited in developing a β-lactam-activated prodrug in which the killing activity is activated within resistant bacteria and by their enzymes. Cephalosporin-ciprofloxacin conjugates, cephalosporin-3′-diazeniumdiolates and cephalothin-Bac8c conjugates are such examples. Many approaches include the development of prodrugs to enable or enhance the oral administration of certain antibiotics, like triclosan glycoside prodrugs, 5-modified 2ʹ-deoxyuridines, tebipenempivoxil, prodrugs of FtsZ-targeting benzamides, avibactam prodrugs and tedizolid phosphate. Prodrugs of carvacrol attained increased solubility and enhanced chemical stability. Some prodrugs were designed to enhance drug uptake by bacteria, like entrobactin-ciprofloxacin (by uptake by iron carriers), and P18 and WMR AMP-prodrugs, which impart selectivity and decreased toxicity. Further to the development of novel prodrugs, the screening for overlooked antibacterial prodrugs presents new potential and a platform for antimicrobial discovery.

## Figures and Tables

**Table 1:** A timeline of antibiotic introduction and the development of antibiotic resistance.

Antibiotics	Date Introduced	Date of Resistance Development
Penicillin	Discovery-1928 Mass production-1942	1940
Tetracycline	1950	1959
Erythromycin	1953	1968
Methicillin	1960	1962
Gentamicin	1967	1979
Vancomycin	1972	1988/2002
Imipenem/Ceftazidime	1985	1998/1987
Levofloxacin	1996	1996
Linezolid	2000	2001
Daptomycin	2003	2014
Ceftaroline	2010	2011
Telavancin	2013	2019
Tedizolid phosphate/Dalbavancin	2014	2016/2015
Delafloxacin	2017	2019
Omadacycline	2018	-
Iclaprim	2019	-
Cefiderocol	2020	-

## References

[1] Gajdacs M. (2019). The Concept of an Ideal Antibiotic: Implications for Drug Design. Molecules.

[2] Ventola C.L. (2015). The antibiotic resistance crisis: Part 1: Causes and threats. Pharm. Ther..

[3] Zaman S.B., Hussain M.A., Nye R., Mehta V., Mamun K.T., Hossain N. (2017). A Review on Antibiotic Resistance: Alarm Bells are Ringing. Cureus.

[4] Karaman R. (2014). Using predrugs to optimize drug candidates. Expert Opin. Drug Discov..

[5] Najjar A., Karaman R. (2019). Successes, failures, and future prospects of prodrugs and their clinical impact. Expert Opin. Drug Discov..

[6] Karaman R. (2013). Prodrugs design based on inter- and intramolecular chemical processes. Chem. Biol. Drug Des..

[7] Najjar A., Karaman R. (2019). The Prodrug Approach in the Era of Drug Design.

[8] Amly W., Karaman R. (2016). Recent updates in utilizing prodrugs in drug delivery (2013–2015). Expert Opin. Drug Deliv..

[9] Dahan A., Khamis M., Agbaria R., Karaman R. (2012). Targeted prodrugs in oral drug delivery: The modern molecular biopharmaceutical approach. Expert Opin. Drug Deliv..

[10] Monserrat-Martinez A., Gambin Y., Sierecki E. (2019). Thinking Outside the Bug: Molecular Targets and Strategies to Overcome Antibiotic Resistance. Int. J. Mol. Sci..

[11] Smyth R.D., Pfeffer M., Van Harken D.R., Cohen A., Hottendorf G.H. (1981). Human pharmacokinetics and disposition of sarmoxicillin, a lipophilic amoxicillin prodrug. Antimicrob. Agents Chemother..

[12] Jusko W.J., Lewis G.P., Schmitt G.W. (1973). Ampicillin and hetacillin pharmacokinetics in normal and anephric subjects. Clin. Pharmacol. Ther..

[13] Jones K.H. (1977). Bioavailability of talampicillin. Br. Med. J..

[14] Soares GM S., Figueiredo L.C., Faveri M., Cortelli S.C., Duarte P.M., Feres M. (2012). Mechanisms of action of systemic antibiotics used in periodontal treatment and mechanisms of bacterial resistance to these drugs. J. Appl. Oral Sci..

[15] Shirley D.A., Heil E.L., Johnson J.K. (2013). Ceftaroline fosamil: A brief clinical review. Infect Dis. Ther..

[16] Kong K.F., Schneper L., Mathee K. (2010). Beta-lactam antibiotics: From antibiosis to resistance and bacteriology. Apmis.

[17] Bush K. (2013). Proliferation and significance of clinically relevant β-lactamases. Ann. N. Y. Acad. Sci..

[18] Cantón R., González-Alba J.M., Galán J.C. (2012). CTX-M enzymes: Origin and diffusion. Front. Microbiol..

[19] Scheld W.M. (2003). Maintaining fluoroquinolone class efficacy: Review of influencing factors. Emerg. Infect. Dis..

[20] Becattini S., Taur Y., Pamer E.G. (2016). Antibiotic-induced changes in the intestinal microbiota and disease. Trends Mol. Med..

[21] Stewardson A.J., Gaïa N., Francois P., Malhotra-Kumar S., Delemont C., de Tejada B.M., Schrenzel J., Harbarth S., Lazarevic V., Wp S. (2015). Collateral damage from oral ciprofloxacin versus nitrofurantoin in outpatients with urinary tract infections: A culture-free analysis of gut microbiota. Clin. Microbiol. Infect..

[22] Evans L.E., Krishna A., Ma Y., Webb T.E., Marshall D.C., Tooke C.L., Spencer J., Clarke T.B., Armstrong A., Edwards A.M. (2019). Exploitation of antibiotic resistance as a novel drug target: Development of a β-lactamase-activated antibacterial prodrug. J. Med. Chem..

[23] Allan R.N., Kelso M.J., Rineh A., Yepuri N.R., Feelisch M., Soren O., Brito-Mutunayagam S., Salib R.J., Stoodley P., Clarke S.C. (2017). Cephalosporin-NO-donor prodrug PYRRO-C3D shows β-lactam-mediated activity against Streptococcus pneumoniae biofilms. Nitric Oxide.

[24] Barraud N., Kardak B.G., Yepuri N.R., Howlin R.P., Webb J.S., Faust S.N., Kjelleberg S., Rice S.A., Kelso M.J. (2012). Cephalosporin-3′-diazeniumdiolates: Targeted NO-Donor Prodrugs for Dispersing Bacterial Biofilms. Angew. Chem. Int. Ed..

[25] Soren O., Rineh A., Silva D.G., Cai Y., Howlin R.P., Allan R.N., Feelisch M., Davies J.C., Connett G.J., Faust S.N. (2019). Cephalosporin nitric oxide-donor prodrug DEA-C3D disperses biofilms formed by clinical cystic fibrosis isolates of Pseudomonas aeruginosa. J. Antimicrob. Chemother..

[26] Collins S.A., Kelso M.J., Rineh A., Yepuri N.R., Coles J., Jackson C.L., Halladay G.D., Walker W.T., Webb J.S., Hall-Stoodley L. (2017). Cephalosporin-3′-Diazeniumdiolate NO Donor Prodrug PYRRO-C3D Enhances Azithromycin Susceptibility of Nontypeable Haemophilus influenzae Biofilms. Antimicrob. Agents Chemother..

[27] Kämpfer P., Rauhoff O., Dott W. (1991). Glycosidase profiles of members of the family Enterobacteriaceae. J. Clin. Microbiol..

[28] Levy C.W., Roujeinikova A., Sedelnikova S., Baker P.J., Stuitje A.R., Slabas A.R., Slabas A.R., Rice D.W., Rafferty J.B. (1999). Molecular basis of triclosan activity. Nature.

[29] Howse G.L., Bovill R.A., Stephens P.J., Osborn H.M. (2019). Synthesis and antibacterial profiles of targeted triclosan derivatives. Eur. J. Med. Chem..

[30] Neumann W., Sassone-Corsi M., Raffatellu M., Nolan E.M. (2018). Esterase-catalyzed siderophore hydrolysis activates an enterobactin–ciprofloxacin conjugate and confers targeted antibacterial activity. J. Am. Chem. Soc..

[31] Zheng T., Nolan E.M. (2014). Enterobactin-mediated delivery of β-lactam antibiotics enhances antibacterial activity against pathogenic Escherichia coli. J. Am. Chem. Soc..

[32] Chairatana P., Zheng T., Nolan E.M. (2015). Targeting virulence: Salmochelin modification tunes the antibacterial activity spectrum of β-lactams for pathogen-selective killing of Escherichia coli. Chem. Sci..

[33] Lai Y., Gallo R.L. (2009). AMPed up immunity: How antimicrobial peptides have multiple roles in immune defense. Trends Immunol..

[34] Hancock R.E., Haney E.F., Gill E.E. (2016). The immunology of host defence peptides: Beyond antimicrobial activity. Nat. Rev. Immunol..

[35] Brogden K.A. (2005). Antimicrobial peptides: Pore formers or metabolic inhibitors in bacteria?. Nat. Rev. Microbiol..

[36] Peschel A., Sahl H.-G. (2006). The co-evolution of host cationic antimicrobial peptides and microbial resistance. Nat. Rev. Microbiol..

[37] Chung P.Y., Khanum R. (2017). Antimicrobial peptides as potential anti-biofilm agents against multidrug-resistant bacteria. J. Microbiol. Immunol. Infect..

[38] Zhou Y., Peng Y. (2013). Synergistic effect of clinically used antibiotics and peptide antibiotics against Gram-positive and Gram-negative bacteria. Exp. Ther. Med..

[39] Samy R.P., Stiles B.G., Franco O.L., Sethi G., Lim L.H. (2017). Animal venoms as antimicrobial agents. Biochem. Pharmacol..

[40] Wang K., Yan J., Chen R., Dang W., Zhang B., Zhang W., Song J., Wang R. (2012). Membrane-active action mode of polybia-CP, a novel antimicrobial peptide isolated from the venom of Polybia paulista. Antimicrob. Agents Chemother..

[41] das Neves R.C., Mortari M.R., Schwartz E.F., Kipnis A., Junqueira-Kipnis A.P. (2019). Antimicrobial and antibiofilm effects of peptides from venom of social Wasp and scorpion on multidrug-resistant Acinetobacter baumannii. Toxins.

[42] Forde É., Shafiy G., Fitzgerald-Hughes D., Strömstedt A.A., Devocelle M. (2018). Action of antimicrobial peptides and their prodrugs on model and biological membranes. J. Pept. Sci..

[43] Hilpert K., Volkmer-Engert R., Walter T., Hancock R.E. (2005). High-throughput generation of small antibacterial peptides with improved activity. Nat. Biotechnol..

[44] Desgranges S., Ruddle C.C., Burke L.P., McFadden T.M., O’Brien J.E., Fitzgerald-Hughes D., Humphreys H., Smyth T.P., Devocelle M. (2012). β-Lactam-host defence peptide conjugates as antibiotic prodrug candidates targeting resistant bacteria. RSC Adv..

[45] Ferrari V., Serpi M. (2015). Nucleoside analogs and tuberculosis: New weapons against an old enemy. Future Med. Chem..

[46] Serpi M., Ferrari V., Pertusati F. (2016). Nucleoside derived antibiotics to fight microbial drug resistance: New utilities for an established class of drugs?. J. Med. Chem..

[47] Khandazhinskaya A.L., Alexandrova L.A., Matyugina E.S., Solyev P.N., Efremenkova O.V., Buckheit K.W., Wilkinson M., Buckheit R.W., Chernousova L.N., Smirnova T.G. (2018). Novel 5′-Norcarbocyclic Pyrimidine Derivatives as Antibacterial Agents. Molecules.

[48] Negrya S.D., Jasko M.V., Solyev P.N., Karpenko I.L., Efremenkova O.V., Vasilyeva B.F., Sumarukova I.G., Kochetkov S.N., Alexandrova L.A. (2020). Synthesis of water-soluble prodrugs of 5-modified 2′-deoxyuridines and their antibacterial activity. J. Antibiot..

[49] McEntee L., Johnson A., Farrington N., Unsworth J., Dane A., Jain A., Cotroneo N., Critchley I., Melnick D., Parr T. (2019). Pharmacodynamics of Tebipenem: New Options for Oral Treatment of Multidrug-Resistant Gram-Negative Infections. Antimicrob. Agents Chemother..

[50] Gordon E.M., Duncton M.A., Gallop M.A. (2018). Orally absorbed derivatives of the β-lactamase inhibitor avibactam. Design of novel prodrugs of sulfate containing drugs. J. Med. Chem..

[51] Moellering R. (2003). Linezolid: The first oxazolidinone antimicrobial. Ann. Intern. Med..

[52] Kanafani Z.A., Corey G.R. (2012). Tedizolid (TR-701): A new oxazolidinone with enhanced potency. Expert Opin. Investig. Drugs.

[53] Barber K.E., Smith J.R., Raut A., Rybak M.J. (2015). Evaluation of tedizolid against Staphylococcus aureus and enterococci with reduced susceptibility to vancomycin, daptomycin or linezolid. J. Antimicrob. Chemother..

[54] Shaw K.J., Poppe S., Schaadt R., Brown-Driver V., Finn J., Pillar C.M., Shinabarger D., Zurenko G. (2008). In vitro activity of TR-700, the antibacterial moiety of the prodrug TR-701, against linezolid-resistant strains. Antimicrob. Agents Chemother..

[55] Kaul M., Mark L., Zhang Y., Parhi A.K., LaVoie E.J., Pilch D.S. (2013). An FtsZ-targeting prodrug with oral antistaphylococcal efficacy in vivo. Antimicrob. Agents Chemother..

[56] Kaul M., Mark L., Zhang Y., Parhi A.K., Lyu Y.L., Pawlak J., Saravolatz S., Saravolatz L.D., Weinstein M.P., LaVoie E.J. (2015). TXA709, an FtsZ-targeting benzamide prodrug with improved pharmacokinetics and enhanced in vivo efficacy against methicillin-resistant Staphylococcus aureus. Antimicrob. Agents Chemother..

[57] Marinelli L., Di Stefano A., Cacciatore I. (2018). Carvacrol and its derivatives as antibacterial agents. Phytochem. Rev..

[58] Marinelli L., Fornasari E., Eusepi P., Ciulla M., Genovese S., Epifano F., Fiorito S., Turkez H., Rtc S., Mingoia M. (2019). Carvacrol prodrugs as novel antimicrobial agents. Eur. J. Med. Chem..

[59] Fleck L.E., North E.J., Lee R.E., Mulcahy L.R., Casadei G., Lewis K. (2014). A screen for and validation of prodrug antimicrobials. Antimicrob. Agents Chemother..

[60] Guay D.R. (2001). An update on the role of nitrofurans in the management of urinary tract infections. Drugs.

[61] Gershon H., Parmegiani R. (1963). Antimicrobial activity of 8-quinolinol, its salts with salicylic acid and 3-hydroxy-2-naphthoic acid, and the respective copper (II) chelates in liquid culture. Appl. Environ. Microbiol..

